# Analysing the effect of paddy rice variety on fluorescence characteristics for nitrogen application monitoring

**DOI:** 10.1098/rsos.180485

**Published:** 2018-06-27

**Authors:** Chaoyong Shen, Zhongke Feng, Daoqin Zhou

**Affiliations:** 1 Beijing Forestry University, Beijing 100083, People's Republic of China; 2 The Third Surveying and Mapping Institute of Guizhou Province, Guiyang 550004, People's Republic of China

**Keywords:** laser-induced fluorescence, nitrogen levels, *Oryza sativa* varieties, multi-variate analysis

## Abstract

Paddy rice is one of the most important cereal crops worldwide, so it is very important to accurately monitor its growth status and photosynthetic efficiency. The nitrogen (N) level is a key factor closely related to crop growth. In this study, laser-induced fluorescence (LIF) technology combined with multi-variate analysis was applied to investigate the effect of paddy rice variety on N fertilizer level monitoring. Principal components analysis was conducted to extract the variables of the main fluorescence characteristics to identify N levels. Experimental results demonstrated that no nitrogen fertilizer can be completely identified for each paddy rice variety. In addition, other N levels can also be well classified based on the fluorescence characteristics. The relationship between the fluorescence ratio (F735/F685 : F735, and F685 denote the fluorescence intensity at 735 nm, and 685 nm, respectively) and leaf N content of different paddy rice varieties is also discussed. Experimental results revealed that LIF technology is an effective method of monitoring the N fertilizer and leaf biochemical components of paddy rice.

## 1. Introduction

Paddy rice (*Oryza sativa* L.) is the main cultivated crop worldwide [1]. Nitrogen (N) is known to be one of the required elements for the growth of paddy rice, so farmers tend to increase the dose of N fertilizer to improve paddy rice yield. However, N fertilizer abuse, which has resulted in serious environmental problems, has attracted much attention throughout the world. Passive remote sensing, which can measure the spectral information of paddy rice, was used as a main method to monitor N levels. Detailed comparative research has also been carried out [2]. However, this technology is closely related to solar radiation, which restricts its application in growth status monitoring. Following this, active remote sensing was widely employed to monitor the growth status of crops. Gong *et al*. [3] proposed hyperspectral light detection and ranging (LiDAR), which can acquire spectral and spatial information from targets with high accuracy. Therefore, hyperspectral LiDAR is used to observe and check the growth status of crops and has been widely studied [4].

Compared with reflectance spectra, investigators found that chlorophyll in leaves can radiate all or part of its absorbed energy at longer wavelengths after being exposed to photons of a certain wavelength—this is known as fluorescence [5]. Related studies have demonstrated that laser-induced fluorescence (LIF) spectra of green plants can be used to monitor plant nutrient deficiencies and species differentiation [6]. These studies have revealed the effects of nutrient deficiency on the fluorescence spectra of corn, and that fluorescence spectral shapes are related to plant types [7,8]. Lichtenthaler & Buschmann [9] analysed the fluorescence spectral characteristics of green leaves and proposed that the fluorescence ratio between red and far-red fluorescence peaks can be used to analyse leaf chlorophyll content. In addition, fast fluorescence kinetics was used to monitor the nutrient stress of crops [10,11]. Thus, chlorophyll fluorescence is a useful tool for vegetation detection.

Several studies have investigated the correlation between fluorescence spectra and leaf N concentration based on LIF technology [12]. Yang *et al.* [13] used passive reflectance and active LIF measurements to study the effect of N fertilization rates on the growth status of corn in a field. They found that leaf chlorophyll concentration displayed a positive correlation with N fertilization rates, as demonstrated by the differences in the fluorescence spectra [13]. Yang *et al.* proposed that the fluorescence characteristics of chlorophyll can be used to monitor nutrient stress in *O. sativa* and emphasized that the far-red fluorescence peak of plants show great potential for monitoring the growth status of plants in the field of remote sensing. Schweiger *et al*. [14] examined the differences in LIF characteristics of leaves of stressed and non-stressed plants and demonstrated that the fluorescence peak ratio between red and far-red fluorescence peaks is applicable to nutrition stress monitoring. In later studies, LIF technology was used as a remote sensing tool to determine the effect of environmental factors (such as light stress, high temperature [15] and drought [16]) on wheat, soya bean and rice. In addition, Yang *et al*. [17–19] investigated the correlation between fluorescence peaks and leaf N content and demonstrated that LIF is a useful technology to monitor the nutrient stress of paddy rice. Živčák *et al*. [20] discussed the effect of different leaf positions on the fluorescence characteristics. However, these investigations have focused on the effects of nutrient stress on the fluorescence characteristics of crops. Investigations into the effects of paddy rice variety on the fluorescence characteristics and the monitoring of leaf N concentration through a combination of LIF technology and multi-variate analysis need to be improved.

Therefore, the main aim of this study was to investigate the effect of paddy rice variety on the fluorescence characteristics and to analyse the performance of fluorescence spectra in the identification of N fertilizer levels through principal components analysis (PCA). In addition, the performance of the fluorescence ratio (F735/ F685 : F735, and F685 denote the fluorescence intensity at 735 and 685 nm, respectively) in monitoring leaf N concentration of different paddy rice varieties is discussed.

## 2. Material and methods

### 2.1. Paddy rice varieties and treatments

Six paddy rice varieties (Manley Indica (V1); Shanyou 63 (V2); Yangliangyou 6 (V3); Y-Liangyou 2 (V4); Victory Indica (V5); II-You 838 (V6)) were cultivated in Xiaogan City, Hubei Province, China, in 2015. The longitude of the experimental area ranges from 113°41′ E to 115°05′ E and the latitude ranges from 29°58′ N to 31°22′ N, which is a typical subtropical monsoon climate. The rainfall and sunshine duration are above 1200 mm and over 1800 h per year, respectively. Four doses of urea N fertilization (N1: 0 kg ha^−1^, N2: 110 kg ha^−1^, N3: 160 kg ha^−1^, N4: 210 kg ha^−1^) were used during the entire growth period. N fertilization was carried out at two different times: 60% at seeding, 40% at tilling. In this area, an absolute block design was used to cultivate each paddy rice variety with three replications under the same cultivation conditions [21]. In addition, other management strategies were performed according to the requirements of the local farm extension service. The leaf samples were taken from second leaves from the top that were fully expanded and were destructively sampled by randomly cutting three leaves with three replicates from three different points in each experimental area. The samples were collected on 1 August 2015.

### 2.2. Fluorescence measurements

The LIF measurement system was built in our laboratory. The excitation source was a 532 nm diode laser with an output power of 450 mW. A telescope was used to collect the excited fluorescence signal, and a single-mode optical fibre with a diameter of 260 µm was used to transmit signals. A 532 nm long-pass filter was placed behind the telescope to eliminate the effect of reflected light from the laser on fluorescence, and the fluorescence signals were measured and recorded.

### 2.3. Leaf nitrogen concentration measurements

The leaf nitrogen concentration (LNC) of paddy rice samples was measured using the Kjeldahl method after the fluorescence spectra were measured [22].

### 2.4. Principal components analysis

Fluorescence spectra with hundreds of wavebands provided higher dimensionality than the number of available samples and included a significant amount of redundant spectral information. PCA, which served as an efficient statistical multi-variate analysis method, is a useful tool to reduce the dimensionality of spectra and extract the most crucial spectral features by analysing the internal correlation [23]. The computed new variables, called principal components (PCs), are then calculated as linear combinations of the original variables [24]. In our study, all fluorescence spectra were analysed through PCA.

## 3. Results

### 3.1. Fluorescence spectra of different paddy rice varieties

**Figure 1.**
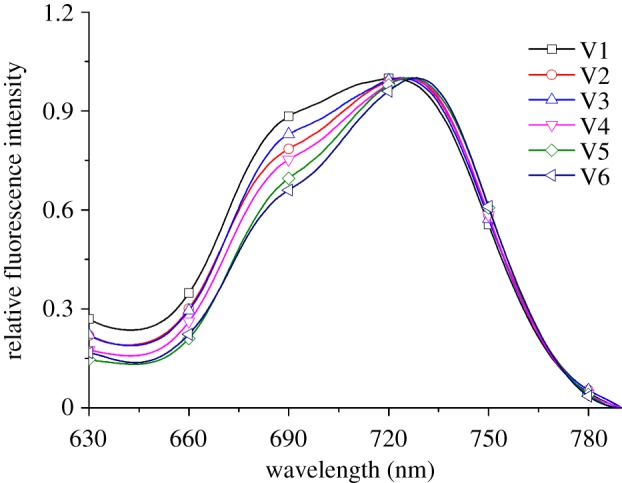
Normalized fluorescence spectra of different paddy rice varieties. (V1) Manley Indica; (V2) Shanyou 63; (V3) Yangliangyou 6; (V4) Y-Liangyou 2; (V5) Victory Indica; (V6) II-You 838.

The normalized fluorescence spectra of six paddy rice varieties are shown in figure 1. The fluorescence spectra range from 630 and 790 nm, wherein the fluorescence peaks are centred at 685 and 735 nm. A related investigation revealed that the fluorescence peak at 685 nm was attributed to chlorophyll *a*, which is associated with photosystem II. The other fluorescence peak at 735 nm corresponded to the antenna chlorophyll of photosystem I and photosystem II [25]. Figure 1 illustrates that the fluorescence intensity excited by 532 nm at 735 nm was higher than that at 685 nm. The possible interpretation for this is that the fluorescence emitted between 684 and 695 nm was more strongly reabsorbed by the chlorophyll pigment than that between 730 and 745 nm in the upper layer leaf cells [26]. Additionally, it can be seen that the fluorescence spectra of different paddy rice varieties exhibited certain differences, possibly because of the effects of reabsorption on different leaf inner structures. However, the reasons for this need to be studied in more detail in the future.

### 3.2. Analysing N levels for different paddy rice varieties

**Figure 2.**
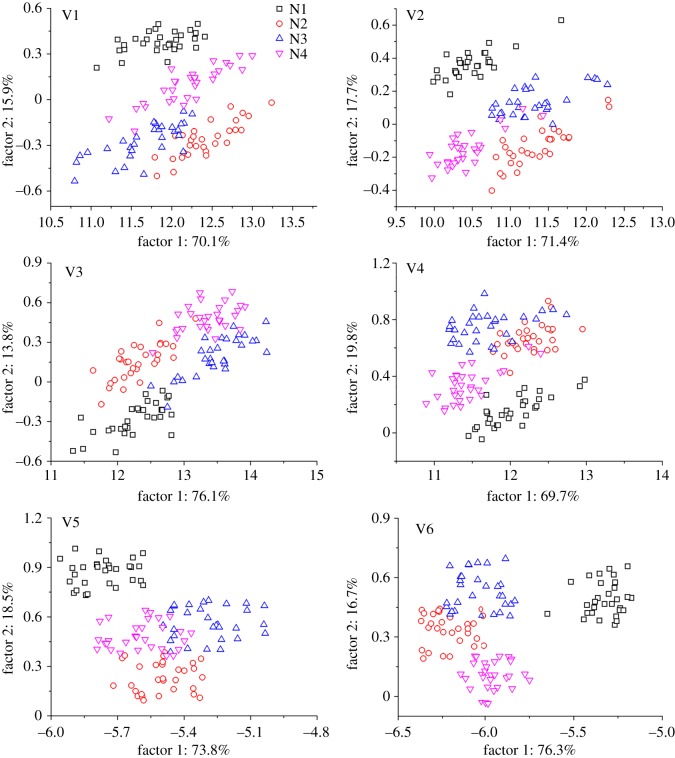
The monitoring of nitrogen levels for different paddy rice varieties by using PCA based on fluorescence spectra. (V1) Manley Indica; (V2) Shanyou 63; (V3) Yangliangyou 6; (V4) Y-Liangyou 2; (V5) Victory Indica; (V6) II-You 838.

The fluorescence spectra of different paddy rice varieties included a large amount of redundant information, which might influence the identification of N levels. Therefore, PCA was used to extract the main fluorescence spectral information and to analyse the dose of N fertilization in this study. When the first two selected PCs could explain the amount of information, and the increase in the explained variance with additional PCs was reduced to less than 2%. Then, 86%, 89.1%, 89.9%, 89.5%, 92.3% and 93% of the total variance contained in the fluorescence spectra can be explained using the first two PCs corresponding to Manley Indica, Shanyou 63, Yangliangyou 6, Y-Liangyou 2, Victory Indica and II-You 838, respectively. Therefore, the first two PCs were used for further discussion, and the two new variables were calculated on the basis of the linear combination of the original variables. Figure 2 shows the scatter distributions and classification rates using the two new variables.

As shown in figure 2, no nitrogen (N1) could be completely distinguished from the other three N levels except for Yangliangyou 6. This finding could be attributed to the close relationship between leaf chlorophyll content and N fertilizer dose, resulting in different fluorescence characteristics [17]. For Yangliangyou 6, the possible reason is that the effect of the inner structure of the leaves on the fluorescence characteristics is more significant than other influencing factors. For the other three N fertilizer levels, the fluorescence characteristics also exhibited certain differences that could be used to identify the growth status of different paddy rice varieties. Thus, the fluorescence characteristics showed a potential for monitoring the nutrition stress of different paddy rice varieties.

### 3.3. Correlation with fluorescence ratio and leaf nitrogen concentration

**Figure 3.**
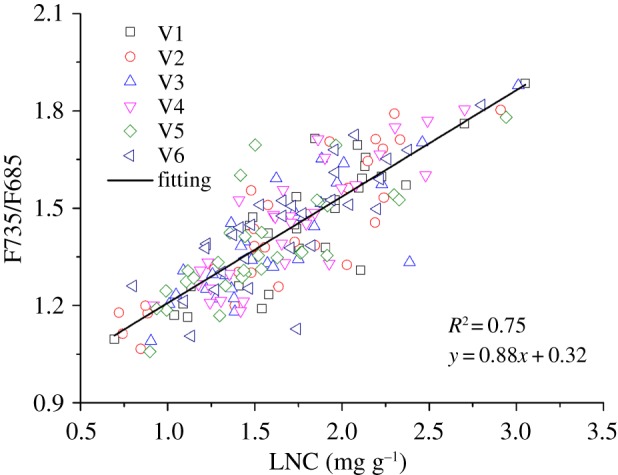
Relationship between the fluorescence ratio (F735/F685) and the LNC of different paddy rice varieties. The solid line is their linear regression. (V1) Manley Indica; (V2) Shanyou 63; (V3) Yangliangyou 6; (V4) Y-Liangyou 2; (V5) Victory Indica; (V6) II-You 838.

The value of the F735/F685 ranges from 1.058 to 1.885, and the mean value is 1.427. The LNC ranges from 0.695 to 3.05 mg g^−1^, and the mean value is 1.670 mg g^−1^ (*n* = 180). The correlation between the fluorescence characteristics (F735/F685) and the LNC of different paddy rice varieties is shown in figure 3.

In figure 3, the fluorescence ratio shows a positive linear correlation with the LNC of all paddy rice varieties. To comprehensively analyse their relationship, a quantitative linear regression analysis between the fluorescence parameters and the LNC for the whole fluorescence dataset was conducted. The linear regression equations *R*^2^ and root-mean-square error of the fluorescence parameters and LNC were 0.75 and 1.808, respectively. The results demonstrate that the relationship between the fluorescence parameters and LNC was highly consistent in all of the samples of different paddy rice varieties, possibly because the fluorescence peak at 685 nm was sensitive to the changes in the leaf biochemical content of plants. Thus, the fluorescence characteristics could be used for the accuracy inversion of LNC.

In this investigation, the performance of LIF technology in the identification of the dose of paddy rice N fertilizer with different varieties was discussed in detail by using PCA. However, only a single-date sample of different paddy rice varieties was used and some limitations should be further considered in subsequent studies. The effects of the different stages of paddy rice growth and the differences in phenology and planting area on the fluorescence characteristics should be investigated to enhance the robustness and reliability of LIF in crop monitoring. In addition, the fluorescence spectra of paddy rice were measured in this study. Subsequent research should consider whole vegetation.

## 4. Conclusion

In this study, fluorescence characteristics were used to analyse N fertilizer application and the LNC for different paddy rice varieties through PCA and linear regression. For different paddy rice varieties, the new fluorescence characteristics extracted based on PCA showed potential for identifying the dose of N fertilizer required. The relationship between the fluorescence ratio (F735/F685) and the LNC was also discussed, and it was found that the fluorescence ratio exhibited a closely positive correlation (*R*^2^ = 0.75) with the LNC for all paddy rice varieties. The experimental results suggested the robustness and reliability of LIF technology for the monitoring of the growth status and biochemical content of paddy rice of different varieties. Thus, LIF combined with multi-variate analysis can be a useful method to estimate the N status and can aid farmers in deciding the correct amount of N fertilizer to apply to crops.

## Supplementary Material

The raw data of fluoescence spectral

## Data accessibility

The datasets supporting this article have been uploaded as part of the electronic supplementary material.
